# The prevalence of medication-related hospital readmissions after bariatric surgery: a retrospective observational study

**DOI:** 10.1007/s11096-025-02017-8

**Published:** 2025-10-06

**Authors:** Jurjen S. Kingma, Cindy Loh, Ingo A. Eland, Marcel P. H. van den Broek, Wouter J. M. Derksen, Catherijne A. J. Knibbe, Fatma Karapinar-Çarkit, Patricia M. L. A. van den Bemt, Desirée M. T. Burgers

**Affiliations:** 1 Department of Clinical Pharmacy, St. Antonius Hospital, Nieuwegein, Utrecht, The Netherlands; 2 Department of Clinical Pharmacy, Hospital Group Twente, Almelo, Hengelo, The Netherlands; 3 Department of Pharmaceutics, Faculty of Science, Utrecht University, Utrecht, The Netherlands; 4 Department of Internal Medicine, St. Antonius Hospital, Nieuwegein, Utrecht, Netherlands; 5 Department of Surgery, St. Antonius Hospital, Nieuwegein, Utrecht, The Netherlands; 6 Division of Systems Pharmacology and Pharmacy, Leiden Academic Centre for Drug Research, Leiden University, Leiden, The Netherlands; 7 Department of Clinical Pharmacy and Toxicology, Maastricht University Medical Centre, Maastricht, The Netherlands; 8 Department of Clinical Pharmacy, CARIM, Cardiovascular Research Institute Maastricht, Maastricht University, Maastricht, The Netherlands; 9 Department of Clinical Pharmacy and Pharmacology, University Medical Centre Groningen, University of Groningen, Groningen, The Netherlands

**Keywords:** Bariatric surgery, Medication errors, Patient readmission

## Abstract

**Introduction:**

Bariatric surgery may induce changes in the effects of oral medication, which may result in medication related problems. Therefore, tailored pharmacotherapy is necessary for individual patients following surgery. In case pharmacotherapy is not adjusted, adverse drug events and even unplanned hospital readmissions may occur. The prevalence of these medication-related readmissions is not known.

**Aim:**

The primary objective of this study was to determine the prevalence of medication-related readmissions within two years after bariatric surgery. Secondary objectives were to determine the percentage of potentially preventable medication-related readmissions. The reasons for readmission, the associated medication, and medication errors involved in the potentially preventable readmissions were also determined.

**Method:**

A retrospective, observational study in a large Dutch teaching hospital was performed. Unplanned readmissions of patients who underwent primary bariatric surgery between January 1, 2018 and August 31, 2020 were included with a follow-up of two years. Records were screened to identify potential medication-related reasons for readmissions. Identified records were independently assessed using adjusted versions of the algorithms of Kramer, Schumock & Thornton for causality and preventability by a doctor and pharmacist. All readmissions assessed as possibly (Kamer scores 0 through 3) or probably (Kramer score 4) related to medication were included. In addition, reasons for readmission, associated medication and medication errors were recorded. Descriptive statistics were used to analyze the data.

**Results:**

In total, 606 unplanned readmissions of 356 individual patients were included. Eighty-three of 606 (13.7%, 95% CI 11.1–16.7%) readmissions were identified as medication-related with a median time between index hospitalization and readmission of 152 days (IQR 16–438). Of these readmissions 36 (43.3%) from 32 unique patients were potentially preventable. The most frequently occurring reasons for readmissions were abdominal pain and infections. Medication most frequently associated with readmissions were Proton Pump Inhibitors, opioids and antibiotics. Medication errors involved in the preventable readmissions were most often prescribing errors followed by non-adherence.

**Conclusion:**

Medication-related readmissions frequently occur after bariatric surgery. More attention is needed for correct pharmacotherapy and patient education in this population.

## Impact statements

- A substantial proportion of medication-related readmissions after bariatric surgery are potentially preventable, indicating an opportunity to optimize pharmacotherapy.
- During the first year after surgery, post-bariatric surgery care should include a structured multidisciplinary medication review and enhanced patient counselling to mitigate the risk of medication-related readmissions.

## Introduction

Medication related problems (MRPs) may lead to patient harm including adverse drug events (ADE) and even hospital admissions. An ADE is harm caused by appropriate or inappropriate use of a drug. This includes events resulting from inappropriate use, such as prescribing errors and non-adherence, as well as adverse drug reactions (defined by the WHO as ‘a response to a medicine which is noxious and unintended, and which occurs at doses normally used in man’) and situations when appropriately used drugs fail to achieve the intended effect [1–3]. ADEs resulting from inappropriate use are potentially preventable. Research showed that 5–19% of all unplanned hospital admissions were medication-related and of these admissions approximately half were potentially preventable [4–8]. The prevalence of medication-related hospital readmissions is even higher and is estimated to be as high as 21–64% within 1 year after the index hospitalization [8, 9]. The preventability of these readmissions was estimated to be 69%. Risk factors of medication-related (re)admissions are age and the number of comorbidities, but also the number of prescribed medications and (for readmissions) the number of medication changes at index admission [7–10].

MRPs may particularly occur within patients undergoing bariatric surgery. Between 2010 and 2030 the prevalence of obesity will increase by 115% to 1.13 billion people [11]. Obesity is associated with comorbidities such as hypercholesterolemia, cardiovascular disease, diabetes and cancer, which lead to higher mortality rates [12, 13]. Currently, bariatric surgery is the most effective treatment for morbid obesity, inducing weight-loss and subsequently reducing comorbidities and mortality [14–16]. The two most commonly performed procedures are the sleeve gastrectomy (SG) and Roux-and-Y gastric bypass (RYGB). Both induce changes in the gastro-intestinal tract which are associated with malabsorption of nutrients [17]. These alterations together with a different eating pattern and weight loss can also induce changes in the pharmacokinetics of orally administered medication caused, for example, by a reduced intestinal surface area or changed gastrointestinal transit time [18]. Therefore it is important to adjust the dose for some medications while other medications need to be avoided. In addition, new medication may be started and monitoring may be required to ensure adequate pharmacotherapy [18–20]. Finally, for some medications, adjustments in the dose may be required while time elapses after bariatric surgery [21]. Of particular relevance in this context is that, during the first year post-surgery, substantial changes in medication regimens are made due to rapid weight loss and subsequent changes in comorbidities [22, 23].

After bariatric surgery, readmission incidence ranges from 5% at 30 days up to 16% at one year after surgery, most often due to gastrointestinal issues related to the surgery leading to abdominal pain [24–26]. The number of medication-related readmissions after bariatric surgery is not known. Insight into this prevalence as well as into the causes and preventability is essential for the development of effective interventions aimed at reducing the risk of medication-related readmissions after bariatric surgery.

### Aim

The primary objective of this study was to determine the prevalence of medication-related readmissions within two years after bariatric surgery. Secondary objectives were to determine the percentage of potentially preventable medication-related readmissions. The reasons for readmission, the associated medication, and medication errors involved in the potentially preventable readmissions were also determined.

## Method

This retrospective observational study was conducted in St. Antonius hospital (Nieuwegein & Utrecht), a large teaching hospital in the central region of the Netherlands. In the St. Antonius Hospital approximately 900 bariatric surgeries are performed each year.

### Study population and unplanned readmissions

Unplanned readmissions at the St. Antonius hospital were eligible for inclusion if the patient had undergone primary bariatric surgery (SG or RYGB) in the St. Antonius hospital between January 1, 2018 and August 31, 2020. The surgery date was registered as the index date with a follow-up period of two years (24 months). This follow-up duration was chosen in order to take into account both short and long term effects on the pharmacokinetics due to bariatric surgery [18, 21]. Only unplanned readmissions were included. An unplanned readmission was defined as an acute hospital visit (e.g. to the emergency room) or acute hospitalisation with overnight stay (for example after an outpatient clinic visit). Unplanned readmissions were excluded if the readmission was due to attempted suicide, if the readmission was to the obstetrics department, if the readmission occurred more than 24 months after index date, or if the readmission took place after revision surgery because this could potentially influence the risk for readmission. Readmissions of patients who objected to sharing data for scientific research were excluded.

### Study procedures

A two-step approach was carried out to identify and subsequently review possible medication-related readmissions. The first step consisted of the identification of potential medication-related hospital readmissions, which was conducted by a trained pharmacy student. For this, an adjusted version of the drug-related hospital admissions (DRA) adjudication guide was used [27]. This is a validated trigger tool for identifying medication-related hospital admissions in older people, which we tailored to the bariatric surgery population, based on clinical experience of the study team, leading to the following adjustments. Within the trigger of acute renal impairment, SGLT2 inhibitors were added. In the trigger of hypocalcaemia diuretic and corticosteroid use were included. Furthermore, abnormal hemoglobin (Hb) levels was added as trigger with the use of iron and vitamin B12 supplementation. We also added abnormal transferrin levels for both iron and multivitamin supplementation use. In addition, deviations in vitamin B12, B1, and B6 levels were added in relation to multivitamin use. Lastly, abnormal folic acid levels were incorporated as potential trigger in patients using multivitamins or antiepileptics.

When the unadjusted trigger tool was used by trained pharmacy students, substantial agreement (81%, κ = 0.62 (CI 0.54–0.70)) between students and an expert panel was shown [28]. If the reason for readmission matched a trigger or event in the trigger tool, the medication overview of the patient at readmission was reviewed, alongside the admission record, lab results and the discharge letter for the presence of suspected causative medication. This information was retrieved from the Hospital Information System (HIS) (EPIC, Systems Corporation, Verona, USA). In case of a match between reason for readmission, trigger or event and suspected causative medication, the case was considered a potential medication-related hospital readmission [27].

The second step consisted of the assessment of causality and preventability. To assess the causality, an adjusted version of the algorithm of Kramer et al. was used, that consisted of the first three axes of the original algorithm [29]. This adjusted algorithm was used in earlier studies on the prevalence of medication-related readmissions [10]. Depending on the score, the causal association was categorised as unlikely (score < 0), possible (score 0 through 3) or probable (score 4). Only readmissions with a possible or probable association with medication were included as medication-related readmissions.

Subsequently, the algorithm of Schumock & Thornton adapted by Lghoul et al. was applied to determine whether the medication-related readmission was preventable [7, 30]. This algorithm consists of ten questions regarding preventable events, which can be answered with either ‘yes’, ‘no’ or ‘too little information to assess’. In order for a medication-related hospital readmission to be labelled potentially preventable, at least one question had to be answered with ‘yes’. This was performed by a hospital pharmacist and a specialist in Internal Medicine. Both assessed the cases independent of each other. In case their assessments differed, consensus was reached. When no consensus could be reached, assessment by a second hospital pharmacist was decisive. When there was more than one reason or medication associated with the readmission, only the most probable (defined as the case with the highest score on the adjusted algorithm of Kramer) was selected for further analysis. In case of a drug-drug interaction, the most recently added medication was selected.

Medication errors involved in potentially preventable readmissions were categorized in the following categories, based on the Systemic Tool to Reduce Inappropriate Prescribing (STRIP) and earlier published research in the bariatric surgery population: undertreatment, dose too low, duplicated side effect (two medications with the same side effect), wrong medication, contra-indication, inadequate monitoring and non-adherence [20, 30, 31]. In order for patients to be categorized as non-adherent to a Proton Pump Inhibitor (PPI), the PPI had to be indicated (in our centre a period of six months post-operatively is customary).

### Outcomes

The primary outcome was the prevalence of medication-related readmissions within two years after primary bariatric surgery (defined as the number of medication-related readmissions divided by the total number of unplanned readmissions). Secondary outcomes were the percentage of potentially preventable medication-related readmissions (defined as the number of potentially preventable medication-related readmissions divided by the total number of medication-related readmissions). The reasons for readmission and the associated medication, and the type of medication error involved in the potentially preventable readmissions were also determined.

### Data collection

Patients were selected from the HIS by the procedural codes for RYGB and SG surgery. All unplanned readmissions in the St. Antonius hospital after the primary surgery were extracted. Data were extracted from the HIS in accordance with standardized protocols and approved hospital procedures, with due consideration for patient privacy and confidentiality. Data were entered in REDCap version 13.1.30 (Vanderbilt University, Nashville, USA) and included the following baseline characteristics at the index admission: sex, age, Body Mass Index (BMI), type of surgery, surgery date, preoperative comorbidities, Charlson Comorbidity Index (CCI) score at index hospitalization [32] and the number of medications after surgery. The following characteristics were collected from the HIS at readmission: age, BMI, readmission and discharge date, readmission department (surgical/non-surgical or visit), length of stay and number of medications at readmission (including dose and regimen). At hospital (re)admission medication reconciliation is routinely performed by trained pharmacy staff before entered in the HIS, and includes both prescription and over-the-counter medications, such as laxatives as well as dietary supplements. If the BMI was not registered in the relevant record, we accepted a previous or future BMI registration of up to four weeks of difference. For the data analysis, the missing BMI values were substituted by the mean BMI of the sample, no other characteristics had missing data. Approximately 10% of data entered in REDcap from the HIS was checked in a random sampling manner. In case an error was observed, an additional 10% of the data was checked until no more errors were encountered. Data were processed in accordance with the General Data Protection Regulation (GDPR).

### Data analysis

Collected data were exported to IBM SPSS Statistics version 26.0.0.1 (IBM, Armonk, USA). Continuous variables were presented as mean with standard deviation (SD) or median with interquartile range (IQR), depending on the distribution. Dichotomous and categorical variables were presented as number and percentage. Comparisons of baseline characteristics between medication-related and medication-unrelated readmissions were made using the appropriate statistical tests. The t-test was used for normally distributed numerical variables and Mann–Whitney U test was used for non-normally distributed numerical variables. For categorical variables, the chi-square test was used or substituted with the Fisher’s exact test when assumptions were not met. The prevalence was calculated using descriptive statistics.

### Ethics approval

The study protocol was reviewed by the Medical Research Ethics Committee (MEC-U) on September 20, 2022 under reference number W22.184, declaring that the study was beyond the scope of the Dutch Medical Research in Humans Act (WMO). Subsequently our local review board reviewed the study protocol and approved the study on September 29, 2022 under reference number Z22.069.

## Results

**Fig. 1:**
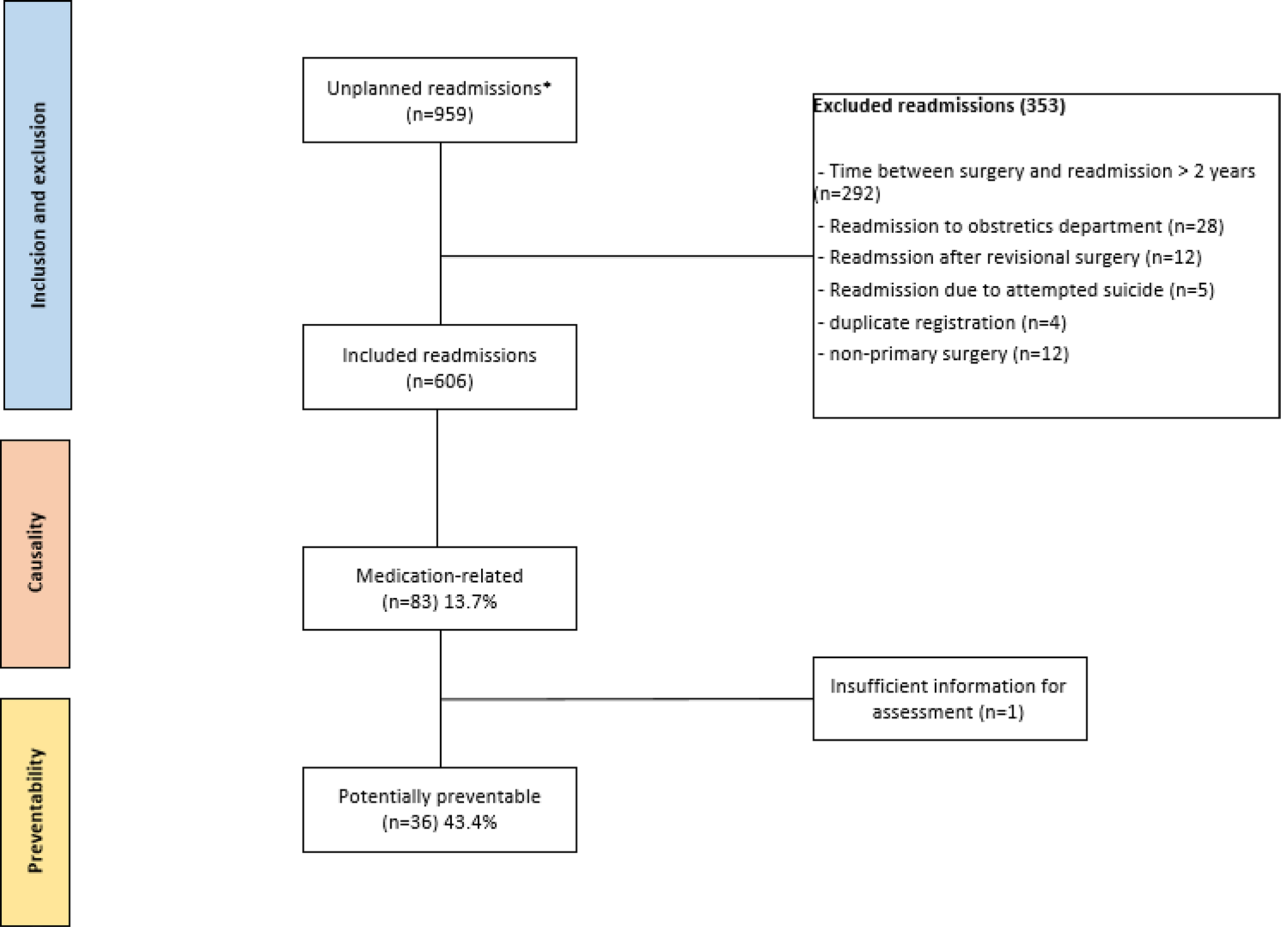
Flow diagram of study procedure. *Unplanned hospital readmissions between 2018 and 2022 of patients who have undergone primary bariatric surgery between January 2018 and August 2020 in the St. Antonius hospital

A total of 959 unplanned readmissions were identified, see Fig. 1. After applying the exclusion criteria, 606 unplanned readmissions from 356 unique patients were eligible, and were assessed for a potential medication-related cause of the readmission, for causality and preventability. Patients were relatively young (mean 44 years) and mostly female (84%). Most common comorbidities were obstructive sleep apnoea, musculoskeletal complaints and hypertension. The median number of medications after bariatric surgery was 5 (IQR 4–8), see Table 1.

**Table 1:** Baseline characteristics of the sample (n = 356 patients) at index hospitalization

Mean age in years (SD)	44.25 (11.75)
Sex, female, n (%)	298 (83.7%)
Mean BMI, kg/m^2^ (SD)	42.58 (4.91)
Comorbidities, n (%):	
Asthma	56 (15.7%)
Hypertension	116 (32.6%)
Hypothyroidism	33 (9.3%)
Musculoskeletal complaints	120 (33.7%)
Obstructive sleep apnoea	229 (64.3%)
Type 2 diabetes	58 (16.3%)
Charlson Comorbidity Index (CCI) score, n (%):	
0 (none)	193 (54.2%)
1–2 (mild)	143 (40.2%)
3–4 (moderate)	18 (5.1%)
> 5 (severe)	2 (0.6%)
Median number of medications after bariatric surgery, (IQR)	5 (4–8)
Type of bariatric surgery, n (%):	
RYGB	232 (65.2%)
SG	124 (34.8%)

*SD* standard deviation, *BMI* Body Mass Index, *IQR* inter quartile range, *RYGB* Roux-en-Y gastric bypass, *SG* sleeve gastrectomy

The mean number of unplanned readmissions per patient was 1.7 ± 1.41. Of the 356 patients, 96.6% had four or less readmissions, there were however some patients with multiple readmissions (n = 4 had five readmissions, n = 1 had six readmissions, n = 2 had seven readmissions, n = 3 had eight readmissions, n = 1 had nine readmissions and one patient had 15 readmissions during the first two years after bariatric surgery). The group of patients with one readmission (n = 225, 63.2%) did not differ from the group with more than one readmission (n = 131, 36.8%) in BMI, sex, age, type of surgery, number of medications after surgery and Charlson Comorbidity Index score (CCI) at index hospitalization.

### Medication-related readmissions

With 83 readmissions assessed as possibly (Kamer scores 0 through 3) or probably (Kramer score 4) related to medication (i.e. medication-related readmission) on a total of 606 unplanned readmissions, the prevalence of potentially medication-related readmissions in the first two years after primary bariatric surgery was 13.7% (95% CI 11.1–16.7%), see Table 2 and Fig. 1. During the first year after surgery, 396 unplanned readmissions occurred of which 58 were medication-related (14.6%) versus 25 medication-related readmissions on a total of 210 unplanned readmissions in year two (11.9%). Patients with medication-related readmissions had a significantly higher median number of medications at time of readmission (6 versus 5 *p* = 0.027). Abdominal pain, followed by infections, bleeding and ulcers were the most commons reason for readmission. Associated medication classes were most often Proton Pump Inhibitors (PPIs), opioids, laxatives and antibiotics, see Table 3.

**Table 2:** Characteristics of patients split for medication-related readmissions (n = 83) and medication unrelated readmissions (n = 523) (total n = 606 unplanned readmissions)

	Medication-related readmission (n = 83)	Medication-unrelated readmissions (n = 523)	*p*-value
Mean age in years (SD)	44.5 (12.3)	44.5 (12.0)	0.978^‡^
Mean BMI, kg/m^2^ (SD)*	34.9 (7.8)	33.1 (7.4)	0.067^‡^
Sex, number of female, (%)	66 (79.5%)	437 (83.6%)	0.363^†^
Median number of days between index hospitalization and readmission, (IQR)	152 (16–438)	247 (39–451)	0.090^‡‡^
Median number of medications, (IQR)	6 (4–9)	5 (3–7)	0.027^‡‡^
Median length of stay, days (IQR)	1 (1–2)	1 (1–2)	0.911^‡‡^
Overnight stay, yes (%)	36 (43.3%)	239 (45.7%)	0.693^†^
Charlson Comorbidity Index (CCI) score at index hospitalization, n (%):			0.161^†^
0 (none)	43 (51.8%)	298 (57.0%)	
1–2 (mild)	34 (41.0%)	194 (37.1%)	
3–4 (moderate)	4 (4.8%)	29 (5.5%)	
> 5 (severe)	2 (2.4%)	2 (0.4%)	
Department of readmission, n (%)			0.720^†^
Emergency	41 (49.4%)	271 (51.8%)	
Surgery	22 (26.5%)	152 (29.1%)	
Emergency cardiac care	4 (4.8%)	11 (2.1%)	
Gastroenterology surgery	4 (4.8%)	31 (5.9%)	
Gastroenterology	3 (3.6%)	17 (3.3%)	
Internal medicine	5 (6.0%)	10 (1.9%)	
Other**	4 (4.8%)	31 (5.9%)	

*SD* standard deviation, *BMI* Body Mass Index, *IQR* inter quartile range

^‡^Independent T-test

^‡‡^Mann–Whitney U test

^†^Pearson Chi-squared when assumptions met, otherwise Fischer Exact test

^*^Missing data were replaced with the mean of sample

^**^Other departments included: cardiology, cardiothoracic surgery, pulmonology, neurology, orthopaedics and urology

**Table 3:** Reasons for medication-related readmissions and potentially preventable medication-related readmissions with the associated medication classes

Reason for readmission	Medication-related readmissions (n = 83)	Potentially preventable medication-related readmissions (n = 36)	Associated medication class (medication-related / potentially preventable medication-related)
Abdominal pain	18 (21.7%)	7 (19.4%)	PPIs (4/1), opioids (4/1), laxatives (4/4), mineral supplements (2/1), digestive enzymes (2/0), antidepressants (1/0), antibiotics (1/0)
Infection	11 (13.3%)	6 (16.7%)*	Antibiotics (8/6), antineoplastics (2/0), antivirals (1/0)
Ulcer	8 (9.6%)	0	PPIs (8/0)
Bleeding	8 (9.6%)	2 (5.6%)	PPIs (3/1), antithrombotics (4/1), antidepressants (1/0)
Severe constipation or fecal impaction	7 (8.4%)	4 (11.1%)	Opioids (4/3), laxatives (2/1), mineral supplements (1/0)
Fall/Fracture	6 (7.2%)	2 (5.6%)	Anxiolytics (2/0), mineral supplements (1/0), antiepileptics (1/0), sedatives (1/1), beta blockers (1/1)
Uncontrolled (non-neuropathic) pain	5 (6.0%)	5 (13.9%)	Opioids (5/5)
Other^‡^	20 (24.1%)	7 (19.4%)	PPIs (4/2), antithrombotics (3/0), antibiotics (3/0), CCBs (2/1), opioids (2/0) vitamins (2/2), hormones (2/1), antipropulsives (1/1), vaccines (1/0)

^*^1 urinary tract infection, 5 skin infections

^‡^Acute renal insufficiency, syncope, anaemia, dehydration, dyspepsia, headache, nervous peroneus neuropathy, Atrioventricular Nodal Reentry Tachycardia (AVNRT), chest pain, electrolyte disturbances, thoracic pain, respiratory disorders. *PPI* Proton Pump Inhibitor, *CCB* Calcium Chanel Blocker

### Potentially preventable medication-related readmissions

Of the 83 medication-related readmissions, 36 readmissions from 32 unique patients, were potentially preventable which corresponds with 43.4% of all medication-related readmissions. Of these 36 medication-related readmissions, 28 occurred in the first year after surgery. Reasons for readmission were abdominal pain, infection, uncontrolled (non-neuropatic) pain and constipation. Opioids were the most common drug class, followed by antibiotics and laxatives, see Table 3. For example, antibiotics were prescribed in a subtherapeutic dosage for skin infections, leading to inappropriate treatment of the infection and subsequent readmission. Another example is opioids prescribed without laxatives, leading to abdominal pain or patients who were already constipated but where not prescribed a laxative. Alternatively opioids were not prescribed despite patients experiencing significant pain, leading to hospital readmission. Also, PPI’s were not taken by the patient, with ulcers as readmission reason.

Of the 36 potentially preventable medication-related readmissions, 28 cases of prescribing errors were identified, i.e. undertreatment n = 16, dose too low n = 6, duplicated side effect n = 3, wrong medication n = 1, inadequate monitoring n = 1 & contra-indication n = 1. In 8 cases, non-adherence errors were identified.

## Discussion

The prevalence of medication-related readmissions in the first two years after primary bariatric surgery is 13.7% of unplanned readmissions, of which 43.4% were potentially preventable. This prevalence was slightly higher in the first year after surgery (14.6%) compared to the second year (11.9%). The most frequently occurring reasons for readmission were abdominal pain and infections, mainly related to non-use of PPIs and laxatives in combination with opioids and the use of antibiotics at an insufficient dosage. The most common reasons for the potentially preventable medication-related readmissions were prescribing errors and non-adherence.

This is the first study investigating medication-related readmissions after bariatric surgery. Although the prevalence of medication-related readmissions is lower than shown in other studies, mainly performed in older persons, where it was estimated as high as 21% within 1 year after the index hospitalization, our population is relatively young (44 years of age) and healthy (Charlson Comorbidity Index score (CCI) none or mild) compared to these study populations [9]. Also, in our study age and CCI was not significantly higher in the medication-related readmissions group compared to the medication-unrelated readmissions while in literature age and CCI are often associated with medication-related readmissions. In our study, PPIs, opioids and antibiotics were most frequently associated with medication-related readmissions. While antibiotics were also identified in other studies, those studies additionally reported associations with antiplatelet agents, anticoagulants, diuretics and antihypertensive medication [8, 33]. The absence of an observed association with diuretics and antihypertensive medication is likely attributable to the fact that these are routinely discontinued postoperatively as part of a standard protocol. In another study investigating risk factors for short-term medication-related readmissions in adults, similar to our findings opioids were identified as a risk factor [34]. Together, these findings suggest that specific medication classes, particularly opioids, PPIs, and antibiotics, may play a key role in medication-related readmissions.

We chose a prolonged follow-up period of two years, which allowed us to study long-term outcomes of bariatric surgery. Within this period, delayed effects of bariatric surgery on the prevalence of medication-related readmissions could be captured, that would otherwise be missed in shorter follow-up studies. We hypothesized that medication-related readmissions required some time to develop, and that earlier readmissions would primarily be related to the surgery itself. However, the prevalence of medication-related readmissions was similar in the first and second year after surgery; of the 396 readmissions in the first year, 58 were medication-related (14.6%) versus 25 medication-related readmissions on a total of 210 readmissions in year two (11.9%). What was notable, however, was that most of the potentially preventable medication-related readmission occurred in the first year after surgery. Also, overall medication-related readmissions occurred earlier than unrelated readmissions, but this difference was not statistically significant.

This study has several limitations. First, this was a single centre study. The dataset is limited to the readmissions at the St. Antonius hospital. Due to the fact that our hospital has a regional function in bariatric surgery, it is conceivable that patients visited other hospitals, possibly leading to an underestimation of the prevalence of medication-related readmissions. The single centre design also limits the generalizability of the results. In our hospital there is more awareness for correct pharmacotherapy in this population due to research conducted earlier and the involvement of clinical pharmacists in the care process [18, 20, 35]. Therefore, the prevalence of medication-related readmissions could differ in other hospitals. A second limitation of this study is the use of an unvalidated screening tool for this population. The screening tool applied in this study, the drug-related hospital admissions (DRA) adjudication guide, is validated for identifying medication-related hospital admissions in older people. Although validated for older persons, other research showed that accuracies of trigger tools were comparable among patient < 70 years of age compared with those > 70 years of age [36]. We adjusted the guide based on our own clinical experiences for our population but this adjusted tool was not validated. This could result in either cases being missed (in which case our prevalence is too low) or cases being selected that are not medication-related. The latter is unlikely, as a thorough assessment followed after the initial screening step. In the study of Coppes et al., the students used discharge letters as their main source [28]. In 31% of the missed medication-related readmissions the information for identifying the readmission was present in the patient records, but not in the discharge letters. In our study the pharmacy student had access to the patient record potentially reducing this percentage. Lastly, no control group was included in this study, which prevents us from concluding that these readmissions are solely attributable to bariatric surgery. For example, the omission of laxatives in patients receiving opioids is a common prescribing error, as demonstrated in other studies [37, 38]. Therefore, further research is warranted in the form of a large, multicentre trial incorporating a well-defined and appropriately matched control group.

This study shows that 43.3% of the medication-related readmissions are potentially preventable, which indicates that improvements in pharmaceutical care may be possible. Most medication errors were prescribing errors and especially concerned undertreatment and dosing errors, which has been reported before in another study from our group [20]. The number of readmissions associated with non-adherence (n = 8) suggests the need for adequate patient education and shared decision making. To address these reasons for readmission, structured medication reviews performed by a multidisciplinary team including pharmacists might improve outcomes [39–41]. Earlier studies showed that involvement of pharmacists leads to a substantial number of interventions in the bariatric surgery population and beneficial impact on enhancing patient adherence [42–48]. Further research should focus on pharmacist-led interventions and medication reviews to reduce the number of readmissions.

## Conclusion

13.7% of unplanned readmissions in the first two years after primary bariatric surgery are medication-related, of which 43.4% were potentially preventable. Of these potentially preventable medication-related readmissions 77.7% occurred in the first year after surgery. Our population was relatively young and healthy, which indicates that bariatric surgery might be a risk factor for medication-related readmissions and that more attention for correct pharmacotherapy is needed. Also, non-adherence was common which suggest that patient education should be improved. Pharmacists play a crucial role in identifying and addressing these issues by informing prescribers but also patients, for example by performing medication reviews [49]. Further research should therefore focus on how the role of pharmacists can be embedded within the care of bariatric surgery patients, particularly in the first year after bariatric surgery.

## Data Availability

The datasets generated and/or analysed during the current study are stored within the hospital, in accordance with privacy regulations, and can be shared with appropriate data sharing agreements upon reasonable request.
